# Anti-tumor effect of innovative tumor treatment device OM-100 through enhancing anti-PD-1 immunotherapy in glioblastoma growth

**DOI:** 10.1038/s41598-024-67437-4

**Published:** 2024-08-08

**Authors:** Zhaoxian Yan, Lifa Huang, Xin Zhang, Xinyan Yu, Rui Huang

**Affiliations:** 1 Academy of Integrative Medicine, Shanghai University of Traditional Chinese Medicine, Shanghai, 201203 China; 2 Department of Neurosurgery, The First Affiliated Hospital of Zhejiang Chinese Medical University, Hangzhou, 310006 Zhejiang China; 3 Department of Medical, Ci Xing Technology Co., Ltd, Hangzhou, 310051 Zhejiang China

**Keywords:** Glioblastoma, Low-frequency magnetic fields, Anti-PD-1 immunotherapy, Innovative tumor treatment device, Apoptosis, Cancer, Cell biology, Immunology, Oncology, Engineering

## Abstract

Glioblastoma (GBM) is associated with a median survival rate of less than 15 months, necessitating innovative treatment approaches. This study investigates the safety and efficacy of the low-frequency magnetic field (LFMF) OM-100 instrument in GBM therapy. In vitro experiments utilized normal astrocyte and GBM cell lines, determining that OM-100 at 100 kHz for 72 h selectively targeted GBM cells without harming normal cells. Subsequent analyses revealed OM-100’s impact on cell viability, apoptosis, migration, invasion, reactive oxide species levels, and PD-L1 expression. In vivo studies on mice with U87-induced GBM demonstrated OM-100's synergy with anti-PD-1 therapy, leading to significant tumor volume reduction and increased apoptosis. Notably, OM-100 exhibited safety in healthy mice. Overall, OM-100 could enhance anti-PD-1 immunotherapy effectiveness probably by directly inhibiting tumor proliferation and migration as well as promoting PD-L1 expression, offering a promising therapeutic strategy for GBM treatment.

## Introduction

Glioblastoma (GBM) is the most common primary brain tumor, typically originating from glial cells in the brain or spinal cord^1^. GBM accounts for nearly 80% of malignant tumors in the central nervous system, with an annual incidence rate of 0.59–5 cases per one hundred thousand^2^. The highly invasive nature of GBM is correlated with a poor prognosis, manifesting as a median overall survival less than 1 year to 14 months for diagnosed patients^3^. Challenges in GBM treatment include incomplete surgical resection, high genetic heterogeneity, the blood–brain barrier presence, and an immunosuppressive microenvironment^1^. Due to resistance to conventional treatment approaches, it remains a great challenge in the oncology field.

Immunotherapy has been a promising avenue for cancer therapy, harnessing the immune system of body to target and eliminate cancer cells^4^. A crucial aspect of this therapy involves disrupting the interaction between programmed cell death protein 1 (PD-1) on immune cells and its ligand PD-L1^5^. This interaction restrains the immune response, allowing cancer cells escape from immune detection and destruction, ultimately leading to resistance to therapy^6^. Immune checkpoint inhibitors, such as anti-PD-1 antibodies, have demonstrated potential in various cancers^7^. Studies by Luo et al. have shown that inducing PD-L1 expression can lead to cell apoptosis and ferroptosis, enhancing the therapeutic effect of anti-PD-1 immunotherapy in non-small cell lung cancer (NSCLC), thus exerting an anti-cancer effect^8^. Rossi et al. have demonstrated the potent therapeutic activity of PD-1 inhibition against GBM^9^. However, the unique microenvironment of GBM and its limited response to immunotherapy call for innovative approaches to improve treatment outcomes.

**Figure 1:**
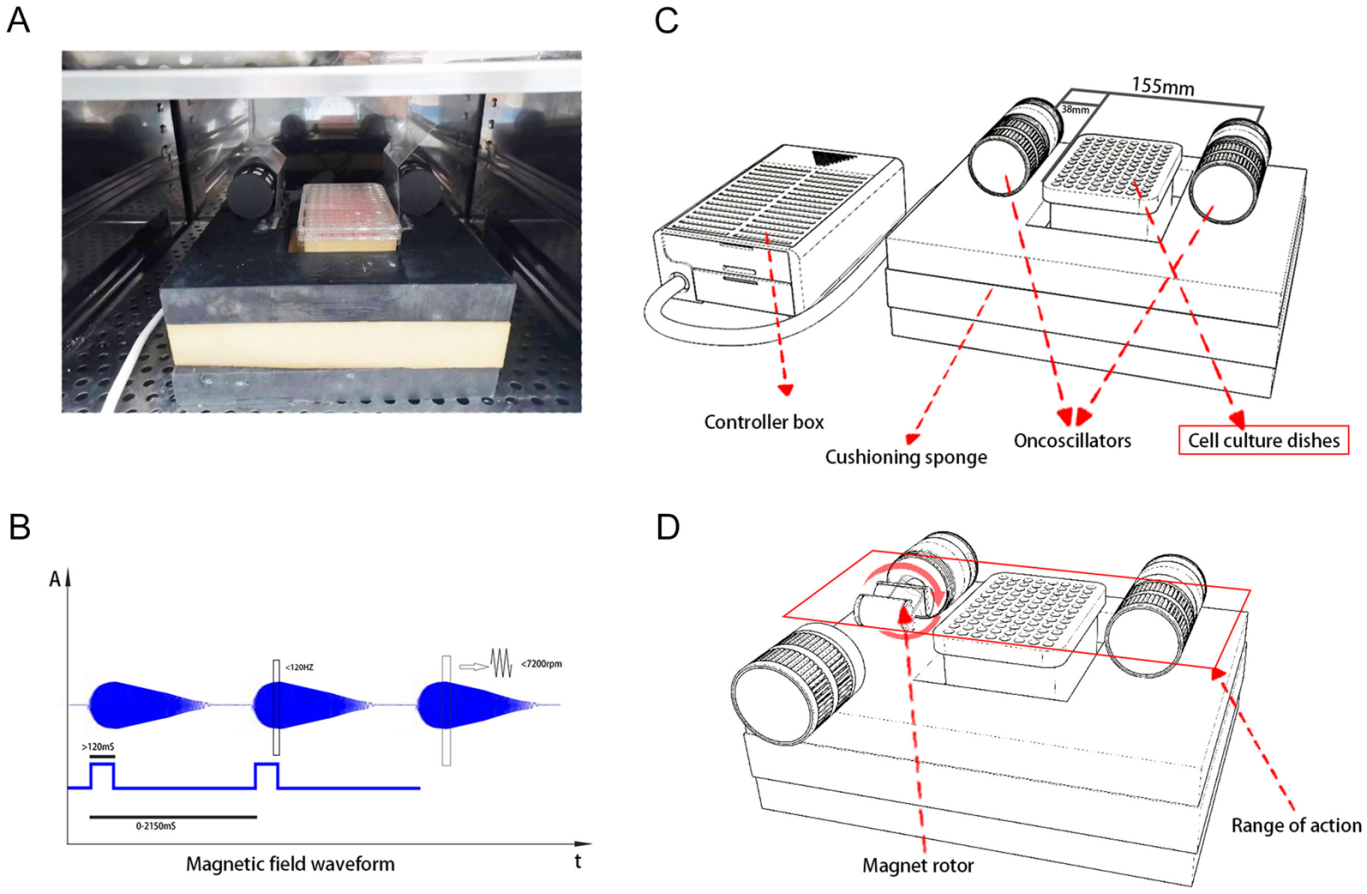
Exposure system and low-frequency magnetic fields (LFMF) of OM-100. (**A**) Photo of the exposure system. (**B**) Magnetic field waveform. (**C**,**D**) Scheme of the exposure system.

Low-frequency magnetic fields (LFMF) are known for their non-invasive, non-ionizing, and non-thermal influences on cells and tissues^10^. Such magnetic fields (MF) can disrupt essential signaling pathways related to cell growth and survival, primarily through interference with ion channels and cell membranes, thereby producing anti-cancer effects^11^. Numerous studies have indicated significant inhibitory effects of LFMF on various cancer, including liver cancer^12^, prostate cancer^13^, and breast cancer^14^, without affecting normal cell growth. Building upon this knowledge, we have developed a novel tumor treatment device named OM-100, equipped with a low-frequency magnetic field ranging from 1.066 to 16.983 mT and a frequency range of 20 to 200 Hz (Fig. 1). Notable features of OM-100 include non-contact operation, absence of heat generation, and precise localization of small lesion areas. It utilizes the low-frequency magnetic field generated by the rotation of high-field magnets to inhibit tumor cell growth. Given that electromagnetic field therapy can be combined with anti-PD-1 therapy to enhance anti-tumor efficacy^15^, we evaluated the safety and efficacy of OM-100.

In this study, we delved into the potential of this innovative tumor treatment device to suppress GBM growth by modulating PD-L1 expression and its role in enhancing the efficacy of anti-PD-1 immunotherapy. The findings represented a novel approach to treating GBM, offering hope for improving the treatment outcomes of this challenging malignancy.

The study is reported in accordance with ARRIVE guidelines (https://arriveguidelines.org).

## Materials and methods

### Cell culture and groups

The human normal astrocyte cell line HA 1800, human GBM cells U87 and U251 were cultured in DMEM with 10% fetal bovine serum and 1% penicillin–streptomycin, and maintained in until reaching a cell density of 80–90% for cell passage. We developed a novel tumor equipment OM-100 which can be used to inhibit the GBM growth by magnetic-field interference. OM-100 was employed to administer treatments at varying durations (0, 24, 48, and 72 h) and different frequencies (0, 10, 25, 50, 100, and 150 kHz) with a magnetic field intensity of 1.066 mT^16^.

### Animal model establishment and grouping

A total of 48 male BALB/c nude mice (6 weeks old, weighing 18–20 g) were procured from the Experimental Animal Center of Yangzhou University. The mice were housed under specific pathogen-free conditions with free access to food and water. They were allowed to acclimate to the environment for one week. Out of these mice, 12 were divided into control and magnetic field treatment groups (n = 6 each). The control group received no treatment, while the magnetic field treatment group underwent a 24-day treatment with a magnetic field intensity of 1.066 mT and a frequency of 100 kHz. After the treatment, changes in body weight and behavioral states of the two groups of mice were observed. Additionally, 24 mice underwent subcutaneous implantation of 100 μL of U87 cells (5 × 10^5^ cells) in the axillary region. When the average tumor volume (TV) of all mice reached approximately 100 mm^3^ (around 6 days), the mice were randomly arranged into four groups, each consisting of 6 mice. The groups included the magnetic field treatment group (GBM + EMF), which was treated with OM-100 for 24 days, and a model group (GBM) as a control. The GBM + anti-PD-1 group and GBM + anti-PD-1 + EMF group after 9 days of magnetic field treatment were treated with anti-PD-1 (10 mg/kg/3 days) by intraperitoneal injection for 15 days.

This study was approved by the Institutional Animal Care and Use Committee of Yangzhou University (Approval No. 202311015), and all animal procedures were performed in accordance with ARRIVE guidelines.

### Cell viability detection

Cell viability in each group was evaluated using the CCK-8 method. Briefly, 10 μL of CCK-8 reaction solution (Beyotime, Shanghai, China) was added to 100 μL of cell suspension treated at different frequencies under a magnetic field intensity of 1.066 mT. After incubation for 2 h, the absorbance was measured by microplate reader (Wuxi Hiwell Diatek, Jiangsu, China).

### Cell apoptosis detection

Flow cytometry was conducted to evaluate cell apoptosis^17^. After enzymatic digestion, cells were subjected to dual staining using Annexin V-FITC binding solution (Beyotime, Shanghai, China) and propidium iodide staining solution.

### Colony formation

After diluting and counting the cell suspension, cells were cultured for 14 days in a CO_2_ incubator (Thermo Scientific, MA, USA). Subsequently, they were stained with crystal violet (Beyotime, Shanghai, China) for 20 min, and the number of clones was then counted.

### Migration assays

For each cell type, three replicates were cultured in 24-well plates with 600 μL of 20% FBS complete medium. After adding 200 μL of cell suspension (1 × 10^5^ cells/mL), cells were incubated for 24 h. Chambers were removed, cells were fixed with methanol (4 °C, 30 min), and stained with crystal violet (20 min). Excess dye was washed with PBS. Three random fields were observed, imaged, and quantified using Image J software (version 1.41a, National Institutes of Health, Bethesda, Maryland, USA, http://rsb.info.nih.gov/ij). Results were processed and visualized with Graphad software (version 8.0, San Diego, California, USA, https://www.graphpad.com/).

### Invasion assays

Matrigel (50 mg/L) was diluted 1:4 (50 μL) on Transwell chamber membranes and gelled at 37 °C for 4 h. Cells (1 × 10^5^/mL) were added to the chambers, and the lower chambers had a medium with 20% fetal bovine serum. After 24 h at 37 °C in a CO_2_ incubator, chambers were removed, cells fixed in 4 °C methanol (30 min), and stained with crystal violet (20 min). Excess cells were wiped away, and chambers were washed thrice with PBS. Cell observations were done in three random fields using a microscope (Olympus, Tokyo, Japan).

### Reactive oxygen species (ROS) detection

Cells were cultured and harvested according to the manufacturer's instructions, and ROS levels were quantified at 450 nm using a fluorescence microplate reader (molecular devices, CA, USA). The fluorescent ROS indicator, DCFH-DA (Solarbio, Beijing, China), was used to label ROS within the cells.

### Western blot analysis

Proteins in myocardial tissue and cells were extracted for Western blotting. The proteins were incubated with primary antibodies at 4 °C overnight, followed by incubation with secondary antibodies. Protein band intensities were visualized using an ECL kit. The primary antibodies used: PD-L1 (1:1000, #PA5-20343, Thermo), GAPHD (1:1000, #2118, CST, MA, USA). For the sary antibody, a Goat Anti-Rabbit IgG H&L (HRP; 1:2000, #ab150077, Abcam, CB, UK) was judiciously employed.

### Flow cytometry analysis

Flow cytometry assessed PD-L1 expression on tumor tissues and cells^18^. Mouse tumor tissues were minced, ground, and filtered through a 300-mesh sieve. Then, 2 mL of cold red blood cell lysis buffer was added. Lysis was stopped with DMEM containing 10% FBS, followed by another centrifugation (300 g, 5 min). After discarding the supernatant, the pellet was resuspended, and the mouse tumor tissue cell suspension was obtained after filtering through a 300-mesh sieve. Cells were trypsinized, centrifuged, and resuspended in 200 μL of PBS. FITC Anti-PD-L1 Antibody (#E-AB-F1133C, Elabscience, Hunan, China) at 5 μL per tube was added, gently mixed, and incubated at room temperature in the dark for 30 min. Afterward, the unbound antibody was removed by centrifugation, and cells were resuspended in 200 μL of PBS for flow cytometry analysis.

### Tumor weight and volume measurements

Every 3 days, tumor volume was measured to observe the dynamic changes in tumor growth. Tumor volume (TV) was calculated using the formula: TV (mm^3^) = 0.5 × a × b^2^, where "a" represents the tumor's maximum diameter, and "b" represents the tumor's minimum diameter. After 24 days of treatment with OM-100, mice were anesthetized with a 10% sodium pentobarbital solution, euthanized, and their tumors were removed, photographed, and weighed.

### Blood routine tests and biochemical indicator analysis

Hematological and biochemical analyses were performed on mouse blood obtained via retro-orbital sampling. A volume of 150 μL of whole blood was mixed with EDTA-K2 anticoagulant. The hematological parameters including granulocytes (Gran), hematocrit (HCT), hemoglobin (HGB), lymphocytes (Lymph), mean corpuscular hemoglobin (MCH), mean corpuscular volume (MCV), platelets (PLT), red blood cells (RBC), mean platelet volume (MPV), and white blood cells (WBC) were measured using an automated hematology analyzer. Subsequently, 650 μL of blood was allowed to stand for 30 min, followed by centrifugation at 10,000 rpm for 2 min. The serum obtained was analyzed for biochemical parameters, including alanine aminotransferase (ALT), aspartate aminotransferase (AST), total bilirubin (T-BIL), creatinine (CREA), triglycerides (TG), total cholesterol (TC), high-density lipoprotein cholesterol (HDL-c), and low-density lipoprotein cholesterol (LDL-c).

### Histopathology examination

Myocardial tissue was examined with hematoxylin–eosin (HE) staining. The left ventricular samples were fixed in 4% paraformaldehyde, embedded in paraffin, and sectioned into approximately 5 μm thick slices. After deparaffinization, the sections were stained with hematoxylin and eosin and examined using a light microscope (Leica, Wetzlar, Germany).

### TUNEL assay

Adopting the experimental protocol by Yu et al.^19^, myocardial tissue sections were treated with proteinase K, followed by the addition of a mixture of TdT enzyme, fluorescent labeling solution, and TUNEL detection solution. Subsequently, a DAPI staining solution (Beyotime) was applied, and the samples were observed under a fluorescence microscope (Olympus, Tokyo, Japan).

### Statistical analysis

The results are presented as Mean ± SD based on three separate experiments. Group differences were detected using a one-way analysis of variance (ANOVA), followed by a post hoc test of Tukey for comparison. Statistical analyses were carried out using GraphPad software (version 8.0, San Diego, California, USA, https://www.graphpad.com/), with statistical significance defined as *P* < 0.05.

### Ethics approval

This study was approved by the Institutional Animal Care and Use Committee of Yangzhou University (number 202311015).

## Results

### OM-100 limits GBM cell growth in vitro

**Figure 2:**
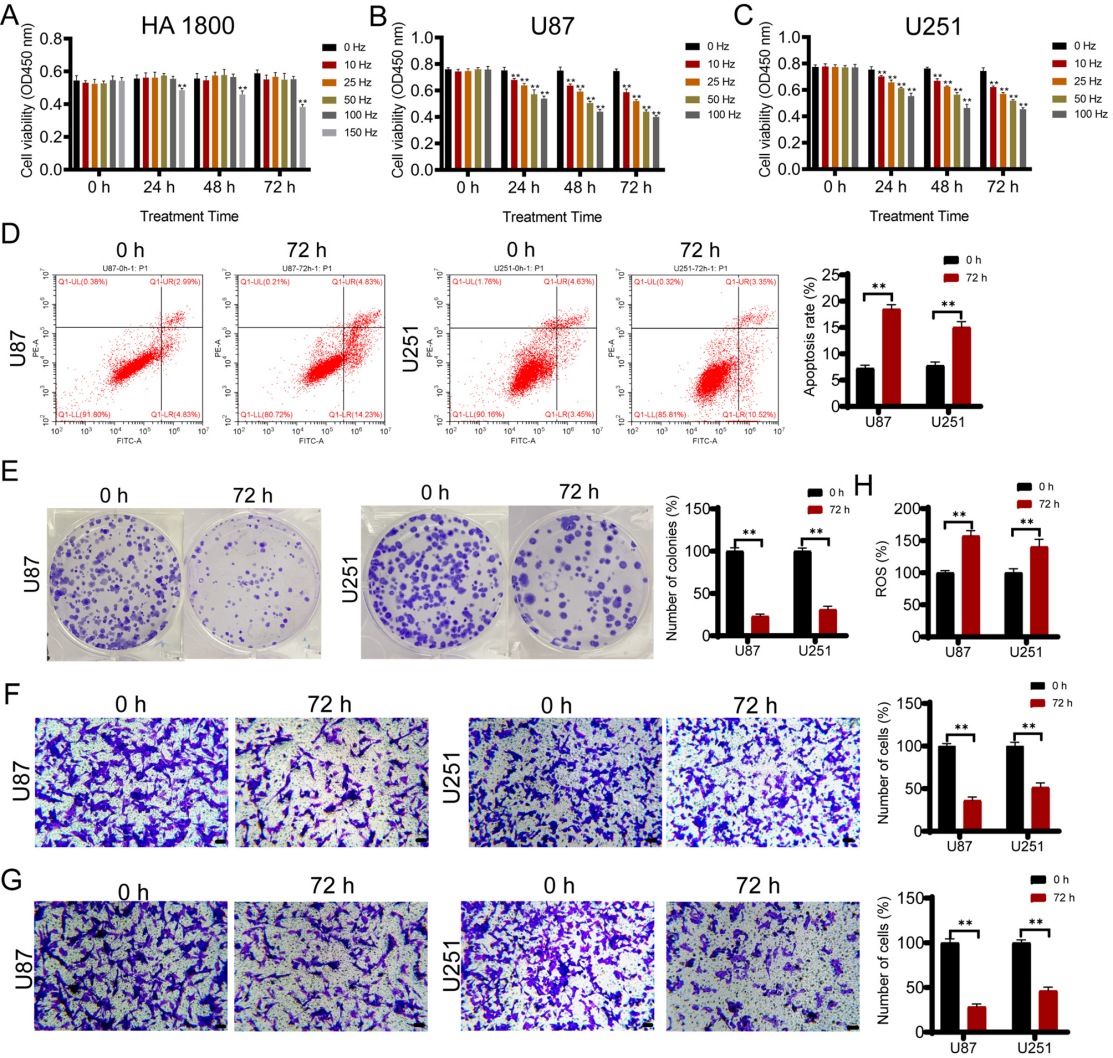
OM-100 limits GBM cell growth in vitro. (**A**) The impact of different frequencies (0 kHz, 10 kHz, 25 kHz, 50 kHz, 100 kHz, and 150 kHz) and durations (0 h, 24 h, 48 h, and 72 h) of OM-100 on the viability of HA 1800. (**B**) The impact of different frequencies (0 kHz, 10 kHz, 25 kHz, 50 kHz, and 100 kHz) and durations (0 h, 24 h, 48 h, and 72 h) of OM-100 on the viability of U87. (**C**) The impact of different frequencies (0 kHz, 10 kHz, 25 kHz, 50 kHz, and 100 kHz) and durations (0 h, 24 h, 48 h, and 72 h) of OM-100 on the viability U251 cell. (**D**)The impact of OM-100 with the condition of 100 kHz and durations (0 h and 72 h) on cell viability of U87 and U251 cells. (**E**) The impact of OM-100 with the condition of 100 kHz and durations (0 h and 72 h) on apoptosis of U87 and U251 cells. (**F**) The impact of OM-100 with the condition of 100 kHz and durations (0 h and 72 h) on migration of U87 and U251 cells. (**G**) The impact of OM-100 with the condition of 100 kHz and durations (0 h and 72 h) on invasion of U87 and U251 cells. (**H**) The impact of OM-100 with the condition of 100 kHz and durations (0 h and 72 h) on migration of U87 and U251 cells. (**G**) The impact of OM-100 with the condition of 100 kHz and durations (0 h and 72 h) on ROS level of U87 and U251 cells. Scale bar = 50 μm. **P < 0.01 when compared with o kHZ. The original protein blot images can be seen in Supplementary file.

To determine the optimal frequency of OM-100, human normal astrocyte cell line HA 1800 was subjected to different frequencies at 1.066 mT magnetic field strength for varying durations. As shown in Fig. 2A, 150 kHz for 24 h, 48 h, and 72 h significantly decreased the cell viability of HA 1800 cells, while 0 kHz, 25 kHz, 50 kHz, and 100 kHz did not significantly affect the viability of HA 1800 cells, regardless of the treatment duration. The results demonstrated that the highest operating frequency for OM-100 targeting normal human cells was 100 kHz. Under identical experimental conditions, a maximum frequency of 100 kHz was applied to human GBM cells U87 and U251. Our observations revealed a significant decline in the viability of both U87 and U251 cells as the magnetic field strength increased (0, 25, 50, and 100 kHz) at 24, 48, and 72 h of treatment (Fig. 2B,C).

Therefore, we selected a frequency of 100 kHz and treated U87 and U251 cells for 0 h and 72 h to evaluate its impact on cell proliferation. Apoptosis detection revealed that the apoptosis rate of U87 and U251 cells was significantly higher after treatment with OM-100 at a frequency of 100 kHz for 72 h than that of untreated cells (Fig. 2D, *P* < 0.01). Additionally, after 72 h of treatment with OM-100, U87 and U251 cells displayed a marked decrease in colony formation after OM-100 treatment compared to the cells that received no treatment (Fig. 2E, *P* < 0.01). The migratory and invasive capabilities of GBM cells were assessed through Transwell assays. Figure 2F–G demonstrated that the cell migration and invasion extents significantly decreased after 72 h of treatment with OM-100 at a frequency of 100 kHz compared to 0 h (*P* < 0.01).

This indicated that OM-100 treatment had an inhibitory effect on the invasive and migratory abilities of cancer cells, which was typically considered a positive indicator of anti-tumor efficacy^20^. Moreover, treatment with OM-100 led to an increase in intracellular ROS levels (Fig. 2H, *P* < 0.01), indicating more oxidation stress in GBM cells. These results indicated the anti-tumor efficacy of OM-100 treatment, which by impairing cell viability, increasing apoptosis, inhibiting cell migration, and invasion capabilities, as well as promoting oxidative stress.

### OM-100 upregulates PD-L1 expression in vitro

**Figure 3:**
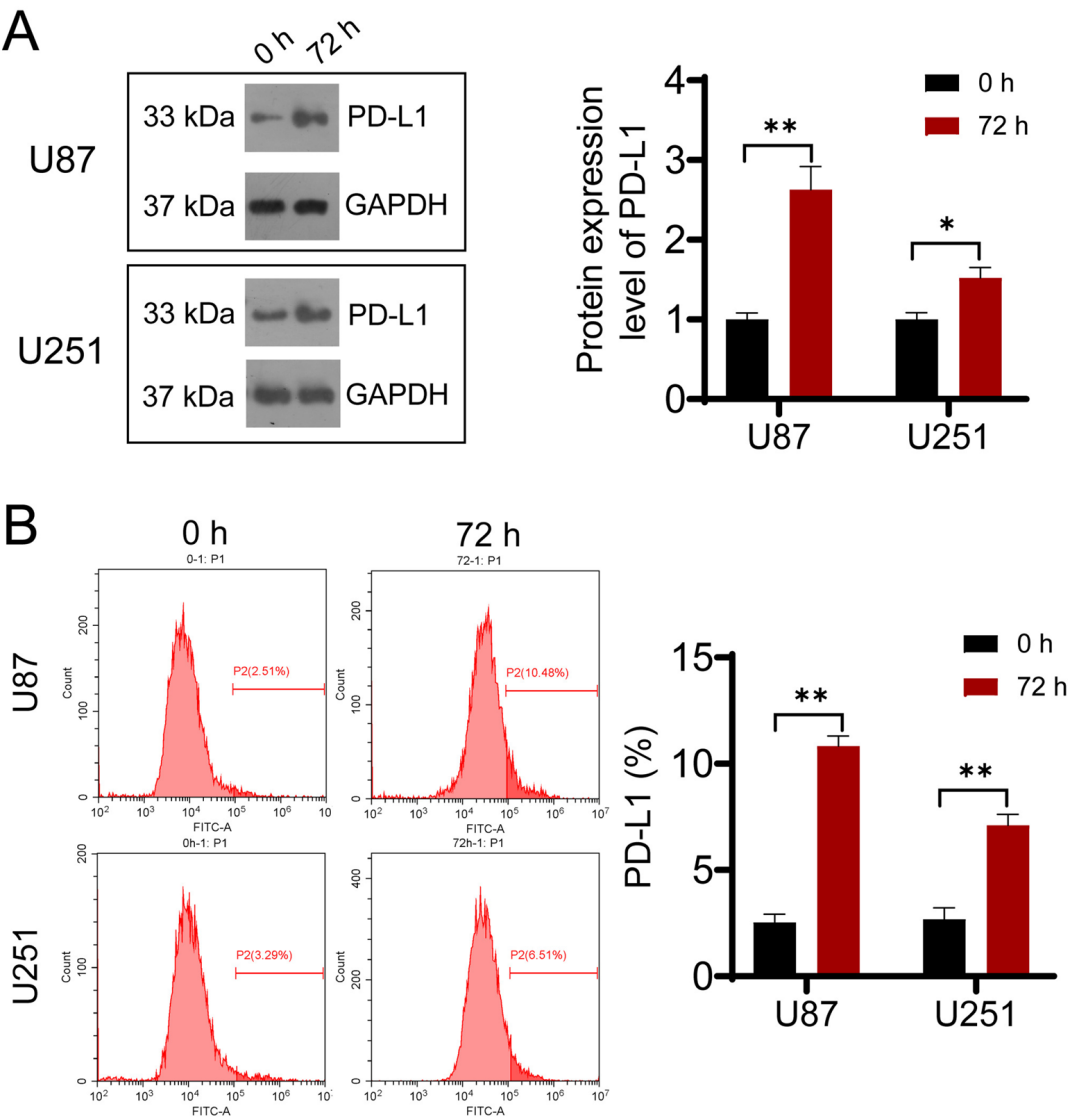
OM-100 upregulates PD-L1 expression in vitro. Western blot (**A**) and flow cytometry (**B**) detected the impact of OM-100 on the expression levels of PD-L1 in U87 and U251 cells. *P < 0.05. **P < 0.01. The original protein blot images can be seen in Supplementary file.

Evidences have revealed the significant correlations across PD-L1 expression and the clinical response of patients who received anti-PD-1 immunotherapy. Specifically, those with PD-L1 + tumor exhibited a more pronounced response to anti-PD-1 therapy in comparison with those with PD-L1- tumors^21^. Further understanding of the regulation of PD-L1 can potentially bring substantial benefits to cancer patients through the enhancement of existing PD-L1/PD-1 blockade therapies. Therefore, we attempted to investigate whether OM-100 might influence PD-L1 expression in GBM cells. Western blot results indicated an upregulation of PD-L1 protein expression undergoing OM-100 treatment (Fig. 3A, *P* < 0.05). Flow cytometry analysis demonstrated that OM-100 could enhance the fluorescence intensity of PD-L1 (Fig. 3B, *P* < 0.01). These findings suggested that OM-100 might potentially impact immune system regulation by upregulating the expression of PD-L1 protein.

### OM-100 inhibits GBM growth by upregulating PD-L1 expression to enhance the efficacy of anti-PD-1 immunotherapy in vivo

**Figure 4:**
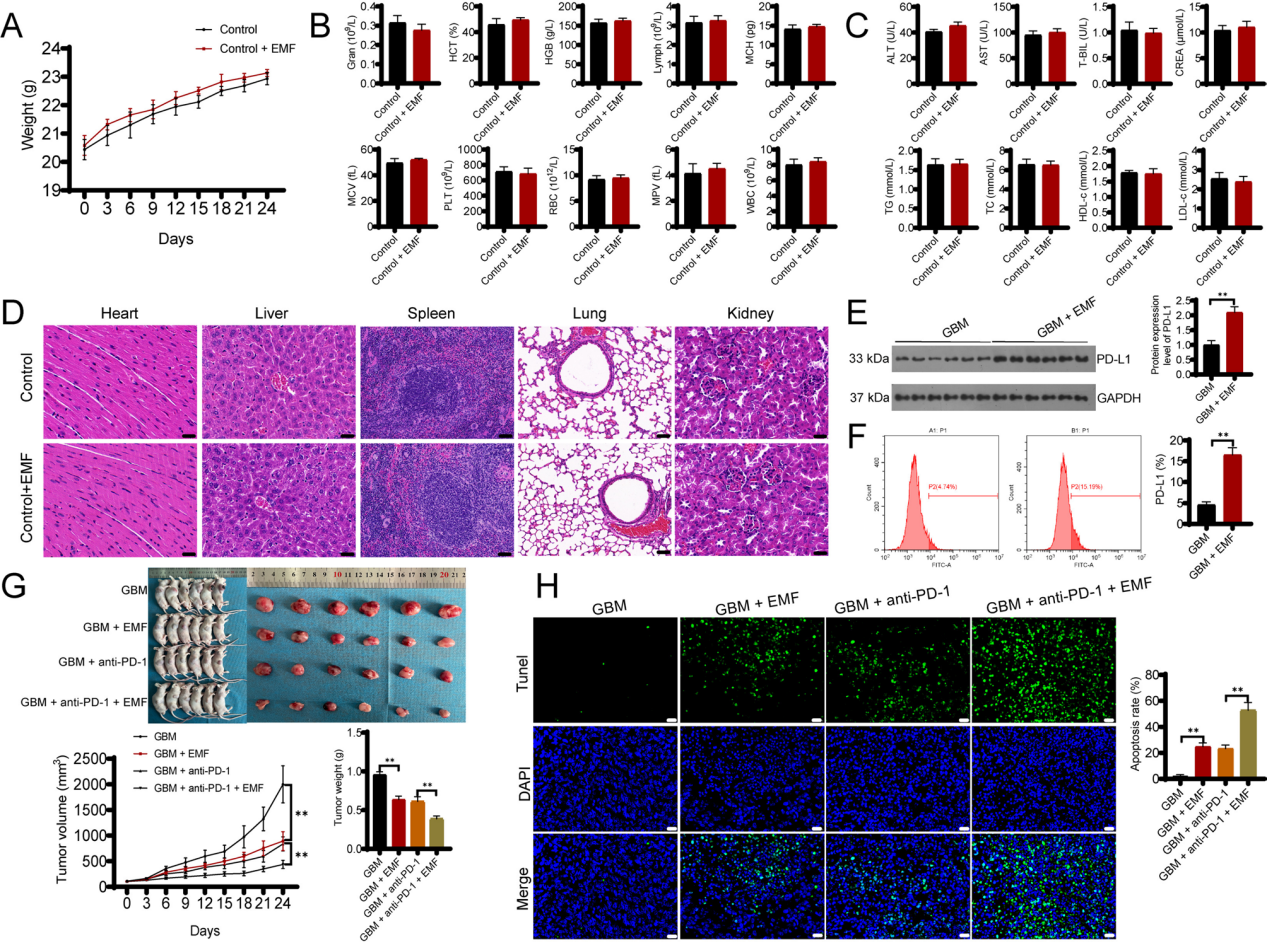
OM-100 inhibits GBM growth by upregulating PD-L1 expression to enhance the efficacy of anti-PD-1 immunotherapy in vivo. The effect of OM-100 on the body weight (**A**), blood routine parameters (granulocytes (Gran), hematocrit (HCT), hemoglobin (HGB), lymphocytes (Lymph), mean corpuscular hemoglobin (MCH), mean corpuscular volume (MCV), platelets (PLT), red blood cells (RBC), mean platelet volume (MPV), and white blood cells (WBC)) (**B**), and biochemical indicators (alanine aminotransferase (ALT), aspartate aminotransferase (AST), total bilirubin (T-BIL), creatinine (CREA), triglycerides (TG), total cholesterol (TC), high-density lipoprotein cholesterol (HDL-c), and low-density lipoprotein cholesterol (LDL-c)) (**C**) of normal mice. (**D**) HE staining was performed to assess cardiomyocytes, liver tissue, spleen tissue, lung tissue, and kidney tissue changes in normal mice affected by OM-100 or not. Western blot (**E**) and flow cytometry (**F**) detected the impact of OM-100 on the expression levels of PD-L1 in glioblastoma tissues. (**G**) Effect of OM-100 on tumor volume and weight size in glioblastoma mice. (**H**) TUNEL detection of apoptotic cell number in glioblastoma tissues. Scale bar = 20 μm. Mice were treated with 1.066 mT of OM-100 at 100 Hz for 24 days. **P < 0.01.

Based on in vitro experiments, we further explored the anti-tumor effects of OM-100 in mice. Initially, we treated healthy mice with OM-100 for 24 days. Following the treatment, the mice displayed excellent well-being, characterized by regular food and water consumption and heightened activity levels. Within 24 days, mice undergoing OM-100 treatment exhibited a gradual increase in body weight, with no significant difference compared to the control group mice (Fig. 4A).

Moreover, application of OM-100 did not result in noteworthy changes in the blood routine parameters (Gran, HCT, HGB, Lymph, MCH, MCV, PLT, RBC, MPV, and WBC) and biochemical indicators (ALT, AST, T-BIL, CREA, TG, TC, HDL-c, and LDL-c) in normal mice (Fig. 4B,C).

HE staining showed that the myocardial tissue of mice before treatment exhibited well-arranged myocardial cells with clear nuclear staining and overall intact cell morphology. Liver tissue displayed hepatic sinusoids radiating between liver cells around the central vein. Spleen tissue exhibited densely packed lymphocytes in the white pulp, with red pulp surrounding it, displaying splenic cords, and the marginal zone had relatively sparse lymphocytes. Lung tissue contained numerous branching bronchioles lined with single-layered epithelial cells. Kidney tissue had abundant vasculature in the renal cortex, containing numerous renal units with an even distribution of renal glomeruli. Histological examination of the heart, liver, spleen, lung, and kidney tissues after treatment did not reveal any significant abnormalities, indicating that OM-100 had no significant impact on the organs of the normal mice (Fig. 4D). However, in tumor-bearing mice within 24 days of treatment using an OM-100 (GBM + EMF group), western blot and flow cytometry results both showed a significant increase in the expression when compared to untreated mice (GBM group) (Fig. 4E,F, *P* < 0.01).

Subsequently, we employed different treatment strategies on GBM-bearing mice. Initially, we used an anti-PD-1 drug (GBM + anti-PD-1 group), followed by a combination treatment with an OM-100 and anti-PD-1 (GBM + anti-PD-1 + EMF group). The results revealed that compared to the control group, both the GBM + EMF group and the GBM + anti-PD-1 group exhibited a significant reduction in tumor volume and weight (Fig. 4G, *P* < 0.01), along with a significant increase in the number of apoptotic cells in tumor tissues (Fig. 4H, *P* < 0.01). Encouragingly, the GBM + anti-PD-1 + EMF group, which received a combined OM-100 and anti-PD-1 treatment, showed even more significant effects with greater reductions in tumor volume and weight compared with the group receiving only the anti-PD-1 drug (GBM + anti-PD-1 group) (Fig. 4G, *P* < 0.01). Additionally, the number of apoptotic cells in tumor tissues was higher in the GBM + anti-PD-1 + EMF group (Fig. 4H, *P* < 0.01). These results indicated that OM-100 could inhibit the growth of GBM by upregulating PD-L1 expression, thereby enhancing the efficacy of anti-PD-1 immunotherapy.

## Discussion

In this study, we highlighted the potential of the novel tumor treatment device OM-100 in enhancing the efficacy of anti-PD-1 immunotherapy in GBM. GBM is notoriously challenging due to its aggressive nature, poor prognosis, and limited response to conventional treatments^22^. Our research on combining LFMF with immunotherapy opened new avenues for GBM treatment. We investigated the effects of OM-100 on GBM both in vitro and in vivo. Results showed OM-100 decreased GBM cell activity, increased oxidative stress, induced apoptosis, reduced cell colonies, inhibited invasion and migration, and promoted PD-L1 expression in vitro. OM-100 enhanced the efficacy of anti-PD-1 immunotherapy by boosting PD-L1 expression in vivo.

In recent years, MF has been used as a non-invasive, safe treatment modality for various diseases^23^. The biological effects of MF on tumor cells depend on various aspects like intensity, frequency, and duration^24^. We found minimal impact on HA 1800 cells at different frequencies, but a significant, time-dependent, and frequency-specific reduction in survival rates for U87 and U251 cells in this study. Furthermore, GBM cells treated with 100 kHz OM-100 induced apoptosis, reduced colony formation, and increased intracellular ROS, highlighting the potential of OM-100 to limit GBM cell growth and invasiveness. Studies indicated that MF can interfere with intracellular ion channels and cell membranes, affecting electrophysiological activities and signaling, and ultimately inhibiting cell growth^11^. MF further affects tumor cell morphology, membrane structure, metabolism, growth, adhesion, immune responses, and microcirculation^24^. In addition, ROS, including superoxides and hydrogen peroxide, play a significant role in tumor progression, metastasis, and apoptosis^25^. LFMF has been shown to significantly increase intracellular ROS levels in various cells^26^. Masoudi-Khoram and Abdolmaleki found that exposing MDA-MB-231 breast cancer cells to 20 mT 50 Hz EMF for 3 h daily over four days decreased cell viability and increased apoptosis rates^27^. These results aligned with our study.

Moreover, the impact of OM-100 on enhancing anti-PD-1 therapy in the anti-tumor process was notably attention-worthy. Immunotherapy, especially anti-PD-1 therapy, has emerged as a promising cancer treatment strategy by blocking the PD-1/PD-L1 axis^28^. However, the unique microenvironment of GBM and limited immunogenicity hinder the efficacy of this approach^29^. In our study, OM-100 demonstrated the ability to upregulate PD-L1 expression in GBM cells, offering a potential solution to this challenge. The upregulated expression of PD-L1 in GBM cells post-OM-100 treatment indicated a potential mechanism whereby the device might modulate immune responses, thereby rendering GBM cells more receptive to anti-PD-1 immunotherapy. Taube et al.^30^ reported that PD-L1 expression was observed on both tumor cells and infiltrating immune cells such as tumor infiltrating lymphocytes and associated histiocytes/macrophages, and its expression differed by tumor type. Particularly, there was a more pronounced response to anti-PD-1 therapy in patients whose tumors expressed PD-L1^30^. Zhang et al.^31^ found that up-regulation of PD-L1 in tumor microenvironment was involved in the amplified anti-PD-1 therapy efficacy of blocking Wnt/ß-catenin signal on GBM. Although PD-L1 is usually considered an immunosuppressive molecule, its expression does not necessarily equate to tumor immune evasion, and it may reflect a sustained anti-tumor immune response, including the production of interferon and other inflammatory factors^32^.

We further evaluated the anti-tumor effects of OM-100 in an in vivo mouse model. Importantly, OM-100 treatment in healthy mice showed no adverse effects, indicating its safety for normal tissues. In mice with GBM, OM-100, either alone or combined with anti-PD-1 therapy, significantly decreased tumor size and weight while increasing apoptosis in tumor tissues. Xu and colleagues also discovered that LFMF inhibited tumor growth and induced autophagic cell death in lung cancer^33^. Notably, the combined treatment of OM-100 with anti-PD-1 therapy showed superior efficacy compared to anti-PD-1 therapy alone. Similarly, both Tumor Treating Fields (TTFields, alternating electric fields used for cancer treatment) and LFMF involve the external application of electromagnetic fields as therapeutic modalities^34,35^. Voloshin et al. hypothesized that the combined treatment of TTFields, with anti-PD-1 therapy led to an increase in PD-L1 density in tumor-infiltrating leukocytes of LLC-1 tumors, likely due to the elevated production of IFN-γ in CD8 + cells within the tumor environment^15^. These findings collectively supported the potential of OM-100 as a complementary therapy to anti-PD-1 immunotherapy in treating GBM. By enhancing PD-L1 expression, OM-100 appeared to sensitize GBM cells to immune attacks, potentially overcoming the unique immunosuppressive microenvironment of GBM (Supplementary Information).

## Conclusion

In this investigation, we found that OM-100 significantly impaired cell activity, increased apoptosis, hindered cell migration, and invasion capabilities, and promoted oxidative stress in GBM cells. Notably, it also enhanced PD-L1 expression no matter in vivo or in vitro, particularly when used in combination with anti-PD-1 immunotherapy. OM-100 was proved to be a potent novel approach for GBM therapy. This study offers renewed hope in improving treatment outcomes and life quality for GBM patients, tackling a persistent challenge in the field of neuro-oncology.

### Supplementary Information

Supplementary Information 1.

Supplementary Information 2.

## Data Availability

The datasets used and/or analyzed during the current study are available from the corresponding author on reasonable request.
