# Supplementary figures and images for: Anti-tumor effect of innovative tumor treatment device OM-100 through enhancing anti-PD-1 immunotherapy in glioblastoma growth
Source: Sci Rep. 2024 Aug 8;14:18444. doi: 10.1038/s41598-024-67437-4 (PMC11310191; doi:10.1038/s41598-024-67437-4)

**Figure 3A**

**U87**

**PD-L1 33 KDa**

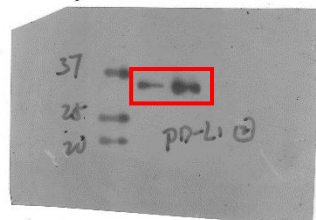

**GAPDH 37 KDa**

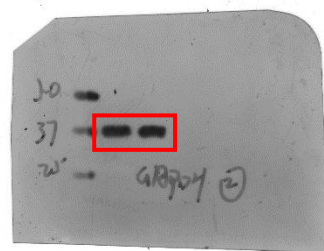

**U251**

**PD-L1 33 KDa**

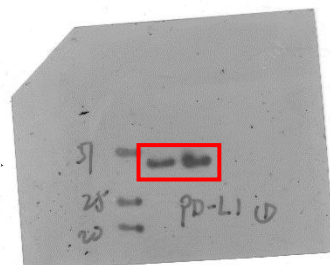

**GAPDH 37 KDa**

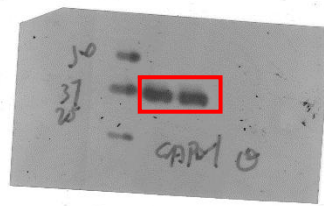

**Figure 4E**

**PD-L1 33 KDa**

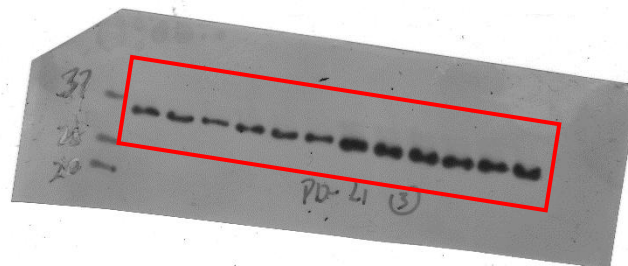

**GAPDH 37 KDa**

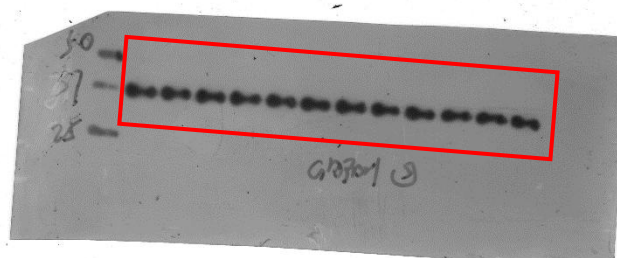

Supplement: Supplementary file 2 — Supplementary Information 2. [file 41598_2024_67437_MOESM2_ESM.pdf]
